# One-year water-ageing of calcium phosphate composite containing nano-silver and quaternary ammonium to inhibit biofilms

**DOI:** 10.1038/ijos.2016.13

**Published:** 2016-06-03

**Authors:** Lei Cheng, Ke Zhang, Chen-Chen Zhou, Michael D Weir, Xue-Dong Zhou, Hockin H K Xu

**Affiliations:** 1 State Key Laboratory of Oral Diseases, West China School of Stomatology, Sichuan University, Chengdu, China; 2 Biomaterials and Tissue Engineering Division, Department of Endodontics, Periodontics and Prosthodontics, University of Maryland School of Dentistry, Baltimore, USA; 3 Department of Orthodontics, School of Stomatology, Capital Medical University, Beijing, China; 4 Center for Stem Cell Biology and Regenerative Medicine, University of Maryland School of Medicine, Baltimore, USA; 5 Department of Mechanical Engineering, University of Maryland, Baltimore County, USA

**Keywords:** antibacterial composite, calcium phosphate nanoparticles, human saliva microcosm biofilm, long-term durability, quaternary ammonium, silver nanoparticles

## Abstract

Dental composites are commonly used restorative materials; however, secondary caries due to biofilm acids remains a major problem. The objectives of this study were (1) to develop a composite containing quaternary ammonium dimethacrylate (QADM), nanoparticles of silver (NAg), and nanoparticles of amorphous calcium phosphate (NACP), and (2) to conduct the first investigation of the mechanical properties, biofilm response and acid production *vs* water-ageing time from 1 day to 12 months. A 4 × 5 design was utilized, with four composites (NACP-QADM composite, NACP-NAg composite, NACP-QADM-NAg composite, and a commercial control composite), and five water-ageing time periods (1 day, and 3, 6, 9, and 12 months). After each water-ageing period, the mechanical properties of the resins were measured in a three-point flexure, and antibacterial properties were tested *via* a dental plaque biofilm model using human saliva as an inoculum. After 12 months of water-ageing, NACP-QADM-NAg had a flexural strength and elastic modulus matching those of the commercial control (*P*>0.1). Incorporation of QADM or NAg into the NACP composite greatly reduced biofilm viability, metabolic activity and acid production. A composite containing both QADM and NAg possessed a stronger antibacterial capability than one with QADM or NAg alone (*P*<0.05). The anti-biofilm activity was maintained after 12 months of water-ageing and showed no significant decrease with increasing time (*P*>0.1). In conclusion, the NACP-QADM-NAg composite decreased biofilm viability and lactic acid production, while matching the load-bearing capability of a commercial composite. There was no decrease in its antibacterial properties after 1 year of water-ageing. The durable antibacterial and mechanical properties indicate that NACP-QADM-NAg composites may be useful in dental restorations to combat caries.

## Introduction

Dental caries is a prevalent worldwide problem,^1^ and tooth cavity restorations cost the United States more than $46 billion annually.^2^ Resin composites are increasingly used as direct filling materials for tooth cavity restorations, because of their excellent aesthetics and improved load-bearing properties.^3, 4, 5, 6, 7, 8^ Extensive studies have significantly improved the microstructure, chemical composition, and physical and mechanical properties of resin composites.^9, 10, 11, 12, 13^ However, dental resin composites tend to accumulate more biofilms/plaques than other restorative materials.^14, 15^ Previous studies have demonstrated that the two main challenges facing composite restorations are secondary caries and bulk fracture.^16^ Replacing the failed restorations accounts for 50%–70% of all tooth cavity restorations performed.^17, 18^ To combat dental caries, antibacterial dental resins have been developed to inhibit oral biofilm acids, which can demineralize the tooth minerals.^19, 20^ The antibacterial resins contain polymerizable quaternary ammonium methacrylates (QAMs), such as 12-methacryloyloxydodecylpyridinium bromide (MDPB), polyethyleneimine (PEI), and quaternary ammonium dimethacrylate (QADM).^21, 22, 23, 24, 25, 26, 27, 28, 29, 30^ In addition, dental composites containing nanoparticles of silver (NAg) have been developed to achieve antibacterial activities.^31, 32, 33, 34, 35^ Incorporating NAg with a high surface area into the resin is desirable to reduce the Ag filler level required for antibacterial efficacy. This approach enables a high antibacterial activity at a low Ag filler level, without compromising the composite colour and mechanical properties.^32, 33^

Another approach to combat caries has been the development of calcium phosphate (CaP)-containing resins.^36, 37, 38^ CaP biomaterials are important because of their excellent biocompatibility, bioactivity, and similarity to minerals in hard tissues.^36, 37, 38, 39, 40, 41, 42^ Composites containing nanoparticles of amorphous calcium phosphate (NACP) release calcium (Ca) and phosphate (P) ions similar to traditional CaP composites, but possess much better mechanical properties.^42, 43^ NACP composites are “smart” and greatly increase the release of Ca and P ions at an acidic pH, at which these ions would be most needed to inhibit caries.^42^ In addition, NACP composites have achieved enamel lesion remineralization rates fourfold higher than that of a fluoride-releasing commercial dental composite.^38^

To further enhance the anti-caries efficacy, the antibacterial approach and the CaP approach have been combined in the same resin, so that the antibacterial agent can reduce biofilms while the Ca and P ions remineralize lesions and inhibit caries.^38, 42, 43^ However, long-term experiments are still needed to investigate the antibacterial durability of the dental resins. A previous study has shown that a composite containing MDPB has a strong antibacterial effect after 3 months of immersion in water.^19^ In another study, the antibacterial activity of a composite containing PEI nanoparticles has been found to be maintained after 1 month of water-ageing.^25^ In addition, the antimicrobial activity of a QAM-containing acrylic resin has been shown to be maintained after 3 months of water-ageing.^44^ However, whether the antibacterial effects decrease after 6 months is unclear, and longer term studies (for example, 1 year) have not been reported for antibacterial dental resins.

Accordingly, the objectives of this study were to develop a bioactive composite containing QADM, NAg, and NACP for the inhibition of caries, and to conduct the first investigation of the mechanical properties, oral biofilm response and lactic acid production as a function of water-ageing time from 1 day to 12 months. We hypothesized that: (1) an NACP composite containing QADM and NAg would have much less biofilm viability and acid production than a commercial composite control; (2) the antibacterial activity of the NACP-QADM-NAg composite would show no significant decrease with increasing water-ageing time from 1 day to 1 year; and (3) the NACP-QADM-NAg composite would possess similar mechanical properties to those of a commercial control composite after a long-term water-ageing treatment.

## Materials and methods

### Synthesis of NACP nanoparticles and composites containing QADM and NAg

NACP (Ca_3_[PO_4_]_2_) were synthesized *via* a spray-drying technique, as described in a previous study.^42^ Briefly, calcium carbonate (CaCO_3_; Fisher, Fair Lawn, NJ, USA) and anhydrous dicalcium phosphate (J.T. Baker, Phillipsburg, NJ, USA) were dissolved in acetic acid to obtain Ca and P ion concentrations of 8 mmol·L^−1^ and 5.333 mmol·L^−1^; respectively, yielding a Ca/P molar ratio of 1.5. This solution was sprayed into the heated chamber of a spray-drying apparatus, and an electrostatic precipitator was used to collect the dried particles. This process produced NACP with a mean particle size of ~116 nm.^42^ NACP consisted of individual particles as well as some clusters.^43^ The clusters contained numerous small particles that were probably stuck together in the spray-drying chamber before the particles were completely dried. Whereas the individual particles had sizes on the order of 10 nm, the clusters had sizes of ~100–300 nm.^43^ Measurement of 100 random particles in a previous study has shown an average size of 37 nm for the individual particles, and an average size of 225 nm for the clusters.^43^

A resin of bisphenol A glycerolate dimethacrylate (BisGMA) and triethylene glycol dimethacrylate (TEGDMA) at 1:1 mass ratio was rendered light-curable with 0.2% camphorquinone and 0.8% ethyl 4-*N*,*N*-dimethylaminobenzoate.^42^ The product is herein referred to as the BisGMA-TEGDMA resin. This resin served as the model system, and the methods of incorporation of NACP, QADM, and NAg could be applied to other dental resin systems.

The synthesis of antibacterial bis(2-methacryloyloxyethyl) dimethylammonium bromide was as previously described.^27, 28, 32^ The bis(2-methacryloyloxyethyl) dimethylammonium bromide was a dimethacrylate, which has been identified as QADM in previous studies.^28, 32^ Briefly, 10 mmol of 2-(*N,N*-dimethylamino)ethyl methacrylate (DMAEMA; Sigma-Aldrich, St Louis, MO, USA) and 10 mmol of 2-bromoethyl methacrylate (BEMA; Monomer-Polymer Labs, Trevose, PA, USA) were combined with 3 g of ethanol in a closed vial. After stirring at 60 °C for 24 h for the reaction to complete, the solvent was removed *via* evaporation under vacuum. This process yielded QADM as a clear and viscous liquid. QADM was mixed with the BisGMA-TEGDMA resin at a QADM mass fraction of 20%. A preliminary study has shown that this mass fraction yields strong antibacterial properties without compromising the resin's mechanical properties. This resin is herein referred to as the BisGMA-TEGDMA-QADM resin.

NAg were formed in a dental resin as described previously.^45^ Briefly, 0.1 g of silver 2-ethylhexanoate (0.1 g) was dissolved into 0.9 g of 2-(tert-butylamino) ethyl methacrylate (TBAEMA; Sigma-Aldrich, St Louis, MO, USA) by gentle stirring. Then, 1% mass fraction of this solution was added to the two resins: the BisGMA-TEGDMA resin and the BisGMA-TEGDMA-QADM resin. This process yielded a 0.1% mass fraction of the Ag salt in each resin. TBAEMA was used because it improved the solubility by forming Ag-N coordination bonds with Ag ions, thereby facilitating Ag salt dissolution in the resin solution. In addition, TBAEMA contains reactive methacrylate groups and can be chemically incorporated into the polymer network upon photo-polymerization.^45^ The 0.1% mass fraction of silver 2-ethylhexanoate in the resin was used in a preliminary study that showed a strong antibacterial activity, without a noticeable colour change to the resin or negative effects on its mechanical properties.

To produce NACP composites, three resins were used: (1) the BisGMA-TEGDMA-QADM resin, (2) the BisGMA-TEGDMA-NAg resin, and (3) the BisGMA-TEGDMA-QADM-NAg resin. Each resin was filled with the NACP and with glass particles for reinforcement (barium-boroaluminosilicate glass with a mean particle size of 1.4 μm; Dentsply Caulk, Milford, DE, USA), which were silanised with 4% 3-methacryloxypropyltrimethoxysilane and 2% *n*-propylamine.^42^ Each resin contained a filler mass fraction of 70%, with 20% of NACP and 50% of glass, which were mixed to yield a cohesive composite paste. Because the resin matrix mass fraction was 30%, the QADM mass fraction in the final composite was 6%.

Therefore, after filling with NACP and glass particles, these three resins resulted in three composites:

1. Composite containing remineralizing NACP and antibacterial QADM (referred to as NACP+QADM);
2. Composite containing NACP and antibacterial NAg (referred to as NACP+NAg);
3. Composite containing NACP, QADM, and NAg (NACP+QADM+NAg).

In addition, a commercial composite served as a comparative control (Renamel Microfill, Cosmedent, Chicago, IL, USA; referred to as the “Control composite”). This resin consisted of fillers of 40 nm to 0.2 micron particle sizes at 60% filler level by volume in a multifunctional acrylic resin of diurethane dimethacrylate and butanediol dimethacrylate. According to the manufacturer, Renamel is indicated for use in Class III, IV, and V restorations.

For mechanical testing, each composite paste was placed into rectangular moulds of 2mm × 2mm × 25 mm. For biofilm testing, disk moulds were used with a diameter of 9 mm and a thickness of 2 mm. All specimens were photo-polymerized (Triad 2000; Dentsply, York, PA, USA) for 1 min on each open side of the mould.

The specimens were immersed in distilled water at 37 °C for 1 day, 3 months, 6 months, 9 months, or 12 months. This constituted a 4 × 5 full factorial design, with four composites and five water-ageing time periods.

### Mechanical testing

The water-aged rectangular specimens were fractured using a computer-controlled Universal Testing Machine (5500R; MTS, Cary, NC, USA) in three-point flexure with a span of 10 mm and a crosshead speed of 1 mm·min^−1^.^42^ Flexural strength (*S*) was calculated as:

*S*=3*P*_max_*L*/(2*bh*^2^)

where *P*_max_ represents the load, *L* represents the span, *b* represents the specimen width and *h* represents the thickness. The elastic modulus (*E*) was calculated as:

*E*=(*P*/*d*)(*L*^3^/[4*bh*^3^])

where load *P* divided by displacement *d* is the slope of the load-displacement curve in the linear elastic region. Six specimens were tested for each composite at each water-ageing time period. The specimens were tested within a few minutes after being taken out of the water, and the specimens were wet while being fractured.

### Human saliva collection for the dental plaque microcosm biofilm model

The dental plaque microcosm biofilm model was approved by the University of Maryland Baltimore Institutional Review Board. This model has the advantage of maintaining much of the complexity and heterogeneity of *in vivo* plaques.^46^ Saliva was collected from 10 healthy adult donors possessing natural dentition without active caries or periopathology and who had not used antibiotics within the previous 3 months.^32, 47, 48^ The donors did not brush their teeth for 24 h and stopped any food/drink intake for at least 2 h before donating saliva. Stimulated saliva was collected during parafilm chewing and kept on ice. An equal volume of saliva from each of the 10 donors was combined to form the saliva sample. The saliva was diluted in sterile glycerol to a saliva concentration of 70%. Aliquots (1 mL) of diluted saliva were stored at −80 °C for subsequent use.^32, 47, 48^

### Bacteria inoculum and live/dead staining of biofilms

Composite disks after water-ageing were sterilized with an ethylene oxide sterilizer (Anprolene AN 74i; Andersen, Haw River, NC, USA) and then degassed, following the manufacturer's instructions. The saliva-glycerol stock was added, using 1:50 final dilution, to a McBain artificial saliva growth medium as the inoculum.^49^ The McBain medium contained mucin (type II, porcine, gastric) at a concentration of 2.5 g·L^−1^; bacteriological peptone, 2.0 g·L^−1^; tryptone, 2.0 g·L^−1^; yeast extract, 1.0 g·L^−1^; NaCl, 0.35 g·L^−1^; KCl, 0.2 g·L^−1^; CaCl_2_, 0.2 g·L^−1^; cysteine hydrochloride, 0.1 g·L^−1^; haemin, 0.001 g·L^−1^; and vitamin K_1_, 0.000 2 g·L^−1^, at pH 7.^49^ Each disk was placed into a well of a 24-well plate. Each well was filled with 1.5 mL of the inoculum, and incubated under 5% CO_2_ at 37 ºC. After 8 h, each disk was transferred to a new 24-well plate with 1.5 mL of fresh medium and incubated under 5% CO_2_ at 37 °C for 16 h. Then, each disk was transferred to a new 24-well plate with 1.5 mL of fresh medium and incubated for 24 h. This totalled 2 days of incubation, which has been shown to be sufficient to form relatively mature biofilms on dental resins.^32, 33^

The composite disks with 2-day biofilms were rinsed with phosphate-buffered saline (PBS) and live/dead stained using the BacLight live/dead bacterial viability kit (Molecular Probes, Eugene, OR, USA).^32, 33, 47, 48^ Imaging was performed *via* a confocal laser scanning microscope (CLSM 510; Carl Zeiss, Thornwood, NY, USA). Four randomly chosen fields of view were photographed for each disk, with six disks per material at each time period, yielding 24 images for each composite at each water-ageing time period.

### 3-[4,5–dimethylthiazol–2–yl]-2,5-diphenyltetrazolium bromide assay of metabolic activity

A 3-[4,5-dimethylthiazol-2-yl]-2,5-diphenyltetrazolium bromide (MTT) assay was used to examine the metabolic activity of the biofilms.^28^ MTT is a colorimetric assay that measures the enzymatic reduction of MTT, a yellow tetrazole, to purple formazan. Disks with 2-day biofilms (*n*=6) were transferred to a new 24-well plate, and 1 mL of MTT dye (0.5 mg·mL^−1^ MTT in PBS) was added to each well and incubated at 37 °C under 5% CO_2_ for 1 h. During the incubation, metabolically active bacteria metabolized the MTT to formazan inside the living cells. Disks were then transferred to new 24-well plates, and 1 mL of dimethyl sulfoxide (DMSO) was added to solubilize the formazan crystals. The plates were incubated for 20 min with gentle mixing at room temperature. Two hundred microlitres of the DMSO solution from each well was collected, and the absorbance at 540 nm was measured *via* a microplate reader (SpectraMax M5; Molecular Devices, Sunnyvale, CA, USA). A higher absorbance indicates a higher formazan concentration, which is related to more metabolic activity in the biofilm on the composite surface.^32, 33^

### Lactic acid production by biofilms adherent on resin disks

Disks with 2-day biofilms (*n*=6) were rinsed with cysteine peptone water (CPW) to remove loose bacteria, and then transferred to 24-well plates containing 1.5 mL of buffered-peptone water (BPW) plus 0.2% sucrose. The specimens were incubated for 3 h to allow the biofilms to produce acid. Then, the BPW solutions were collected for lactate analysis using an enzymatic method.^32, 33^ The 340-nm absorbance of BPW was measured with a microplate reader (SpectraMax M5; Molecular Devices, Sunnyvale, CA, USA). Standard curves were prepared using a standard lactic acid (Supelco Analytical, Bellefonte, PA, USA) as described in previous studies.^32, 33^

### Colony-forming unit counts of biofilms adherent on composite disks

Disks with 2-day biofilms were transferred into tubes with 2 mL CPW, and the biofilms were harvested by sonication and vortexing *via* a vortex mixer (Fisher, Pittsburgh, PA, USA), by following the methods used in previous studies.^32, 33^ Three types of agar plates were prepared. First, tryptic soy blood agar culture plates were used to determine the total number of microorganisms.^32, 33^ Second, mitis salivarius agar (MSA) culture plates, containing 15% sucrose, were used to determine the total number of streptococci.^32, 33, 50^ This count was feasible because MSA contains the selective agents crystal violet, potassium tellurite, and trypan blue, which inhibit most Gram-negative bacilli and most Gram-positive bacteria except streptococci, thus enabling streptococci to grow.^50^ Third, cariogenic mutans streptococci are known to be resistant to bacitracin, and this property is often used to isolate mutans streptococci from the highly heterogeneous oral microflora. Hence, MSA agar culture plates plus 0.2 units of bacitracin per mL were used to determine the number of mutant streptococci.^32, 33, 51^ The bacterial suspensions were serially diluted and spread onto agar plates for colony-forming unit (CFU) analysis by following the methods used in previous studies.

### Statistical analysis

Statistical analyses were performed using Statistical Package for the Social Sciences (SPSS 19.0; SPSS, Chicago, IL, USA). Two-way analyses-of-variance (ANOVA) were performed to detect the significant effects of the variables. Tukey's multiple comparison was used to compare the data at a *P-*value of 0.05.

## Results

**Figure 1:**
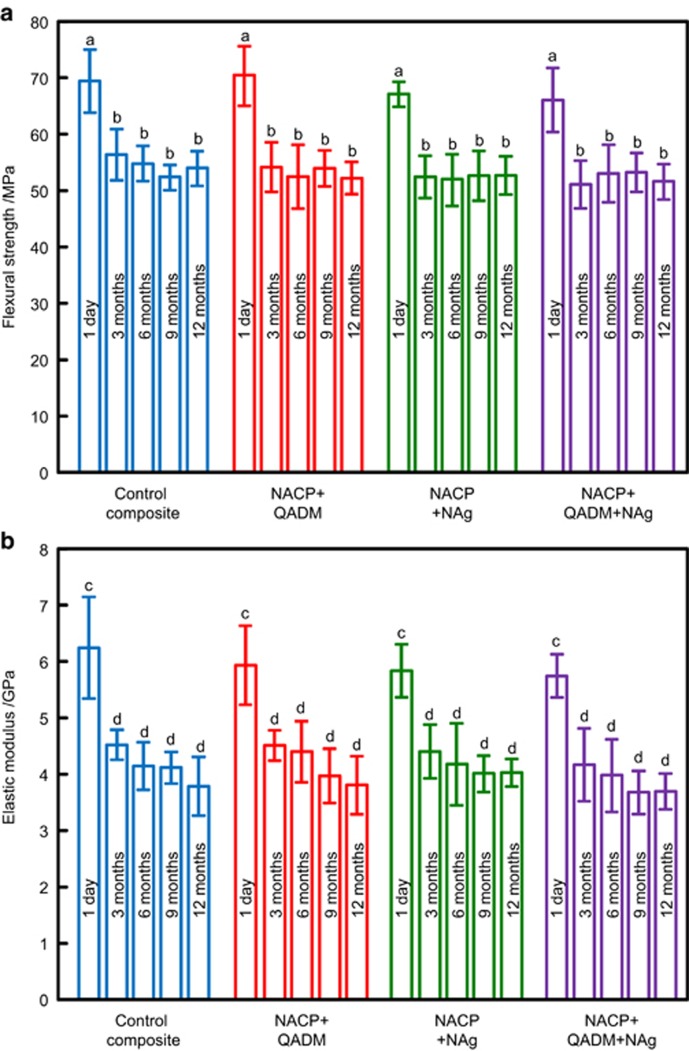
**Mechanical properties of composites during water-ageing.** (**a**) Flexural strength; (**b**) elastic modulus. Each value represents the mean of six measurements, with the error bar indicating one standard deviation (mean±standard deviation; *n*=6). In each plot, values with dissimilar letters are significantly different from each other (*P*<0.05). There was a moderate decrease in the first 3 months, with little subsequent decrease from 3 to 12 months. At 12 months, the NACP-QADM-NAg composite had mechanical properties similar to those of the commercial control composite without antibacterial or remineralizing properties (*P*>0.1). NACP, nanoparticles of amorphous calcium phosphate; NAg, nanoparticles of silver; QADM, quaternary ammonium dimethacrylate.

The flexural strength and elastic modulus results are plotted in Figure 1 (mean±standard deviation; *n*=6). For all four composites, there was a moderate decrease in strength and modulus from 1 day to 3 months of water-ageing (*P*<0.05). There was little subsequent decrease from 3 to 12 months (*P*>0.1). At each water-ageing time period, the flexural strength and elastic modulus of NACP-QADM, NACP-NAg, and NACP-QADM-NAg composites were similar to those of the commercial control composite (*P*>0.1).

**Figure 2:**
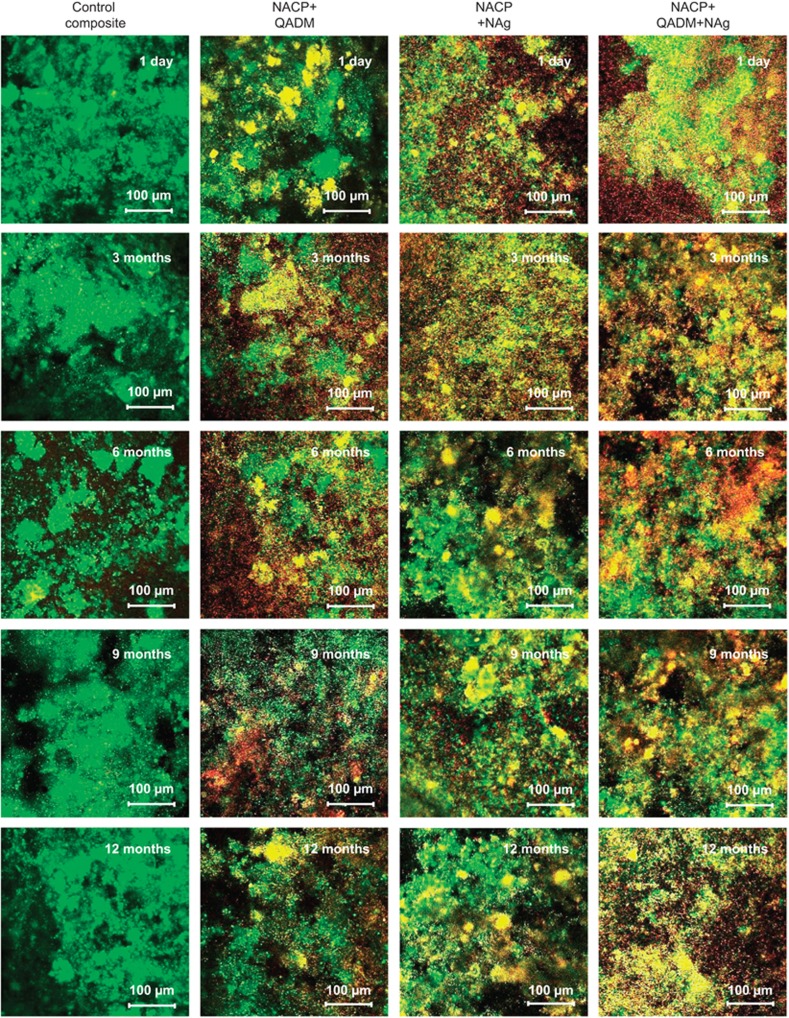
**Live/dead staining of 2-day dental plaque microcosm biofilms on the composites.** Live bacteria were stained green, and dead bacteria were stained red. Live and dead bacteria in proximity resulted in yellow/orange colours. The control composite was covered by a thick biofilm with green staining. NACP composites containing QADM and NAg had much more dead bacteria. There was little difference in biofilm appearance *vs* ageing time, indicating that the antibacterial activity was not lost during water-ageing. NACP, nanoparticles of amorphous calcium phosphate; NAg, nanoparticles of silver; QADM, quaternary ammonium dimethacrylate.

Representative live/dead images of 2-day biofilms on the composites are shown in Figure 2. Live bacteria were stained green, and dead bacteria were stained red. When the live and dead bacteria were in proximity, the biofilm was co-stained with the two fluorophores, resulting in yellowish or orange colours. The commercial control composite disks were completely covered by a continuous biofilm with green staining. In contrast, the antibacterial NACP+QADM, NACP+NAg, and NACP+QADM+NAg composites had much more red and yellow/orange staining colours. For each composite, after water-ageing for 3, 6, 9, or 12 months, there was no noticeable difference in the biofilms compared with that at 1 day of water-ageing. These results indicate that the antibacterial activity of NACP+QADM, NACP+NAg, and NACP+QADM+NAg composites was durable and not lost during the water-ageing for 12 months.

**Figure 3:**
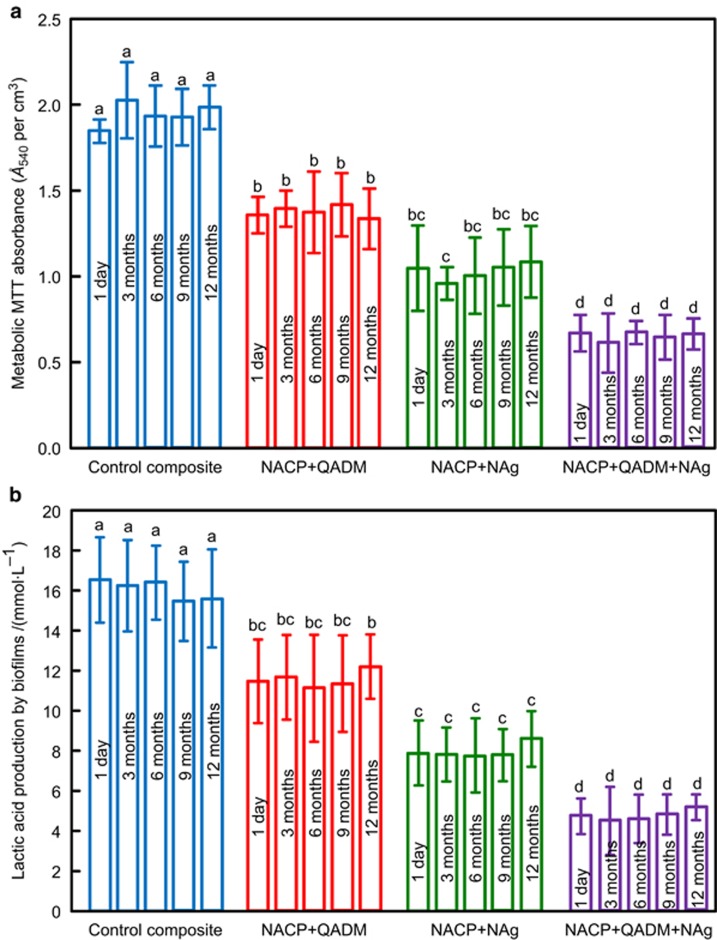
**Quantification of biofilm viability.** (**a**) MTT metabolic activity; (**b**) lactic acid production of 2-day biofilms on the composites after being water-aged for 1 day to 12 months. Each value represents the mean±standard deviation; *n*=6. In each plot, values with dissimilar letters are significantly different from each other (*P*<0.05). Water-ageing for 12 months did not reduce the antibacterial properties of the composite containing QADM and NAg (*P*>0.1). MTT, 3-[4,5–dimethylthiazol–2–yl]-2,5-diphenyltetrazolium bromide; NACP, nanoparticles of amorphous calcium phosphate; NAg, nanoparticles of silver; QADM, quaternary ammonium dimethacrylate.

Figure 3 shows plots of the quantitative biofilm viability results: (a) metabolic activity measured via the MTT assay and (b) lactic acid production of 2-day biofilms on composite disks. The NACP composites containing QADM or NAg yielded much lower metabolic activity and lactic acid production than the commercial composite (*P*<0.05). Furthermore, incorporating QADM and NAg together into the same composite resulted in the lowest metabolic activity and lactic acid production (*P*<0.05). For all composites, the biofilm metabolic activity was similar from 1 day to 12 months of water-ageing (*P*>0.1), and the lactic acid production from 1 day to 12 months was also not significantly changed over this period (*P*<0.05). These results demonstrate that incorporating QADM and NAg into the NACP composite substantially decreased the biofilm metabolic activity and lactic acid production, and the antibacterial potency was maintained and durable during water-ageing for 1 year.

**Figure 4:**
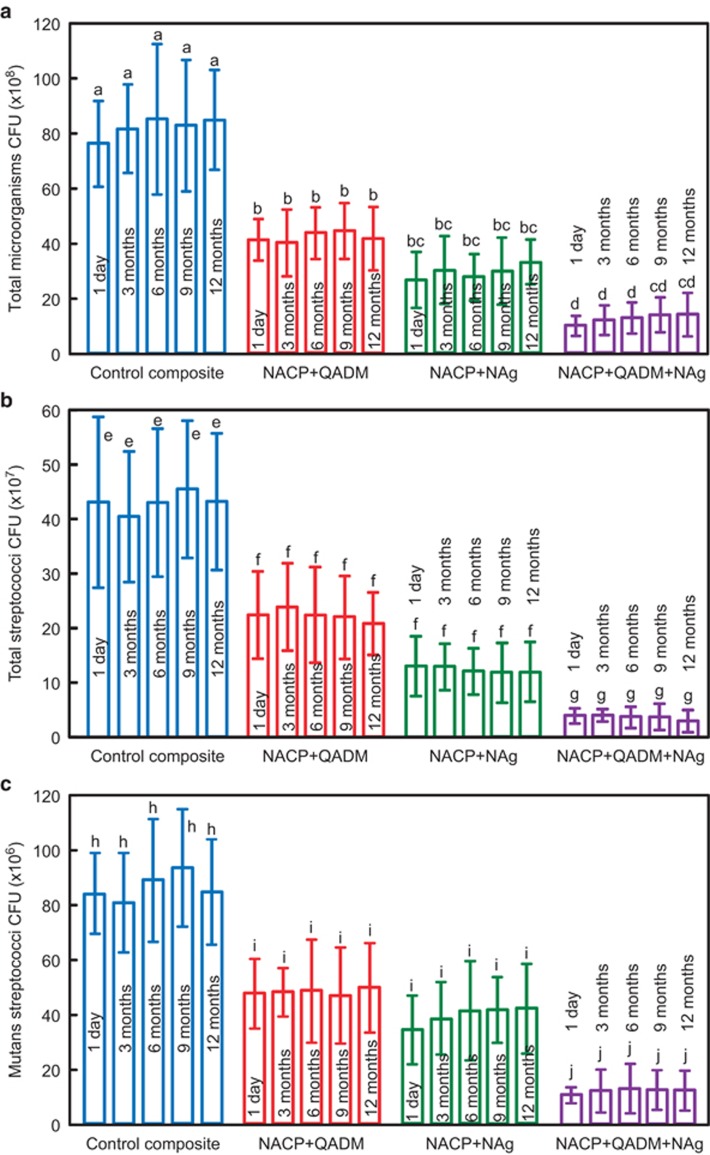
**CFUs of 2-day biofilms on composites after water-ageing for 12 months.** (**a**) Total microorganisms; (**b**) total streptococci; (**c**) mutans streptococci. Combining QADM and NAg together in NACP composite achieved a stronger antibacterial activity than using QADM or NAg alone. Water-ageing for 12 months did not reduce the antibacterial potency of composite containing QADM and NAg (*P*>0.1). Each value represents the mean±standard deviation; *n*=6. Values with dissimilar letters are significantly different from each other (*P*<0.05). CFU, colony-forming unit; NACP, nanoparticles of amorphous calcium phosphate; NAg, nanoparticles of silver; QADM, quaternary ammonium dimethacrylate.

The CFU counts for 2-day biofilms on composite disks are plotted in Figure 4, including (a) total microorganisms, (b) total streptococci, and (c) mutans streptococci (mean±standard deviation; *n*=6). Adding QADM or NAg significantly reduced the CFUs compared with the commercial composite control, and combining both QADM and NAg into the same composite yielded the least CFU counts. For each composite, the CFU counts of biofilms did not vary significantly vs water-ageing time from 1 day to 12 months. For example, the NACP-QADM-NAg composite had a similar number of CFUs at 1 day and after 3, 6, 9, or 12 months of water-ageing (*P*>0.1).

## Discussion

In the present study, antibacterial and remineralizing dental composites were developed containing QADM, NAg, and NACP, which were demonstrated to maintain the anti-biofilm activity after 12 months of water-ageing. Incorporating QADM and/or NAg into the NACP composite did not decrease its mechanical properties, which matched those of a commercial composite control after water-ageing for 1 year. Quaternary ammonium salts are important compounds with a broad antimicrobial spectrum. The advantage of QAM-containing dental resins is that the antibacterial monomer is copolymerized with the resin matrix by forming a covalent bond with the polymer network, and therefore, the QAM is immobilized in the resin and is not released or lost over time. Therefore, this copolymerization and immobilization method may impart a durable antibacterial capability to the dental resin. The present study provides the first demonstration that after 1 year of water-ageing, the antibacterial potency of a composite containing QADM, NAg, and NACP is still as strong as it was at 1 day. Such sustained reduction in biofilm/plaque and acid production may help minimize tooth structure demineralization, and the addition of NACP may promote remineralization.^38^

The present study used QADM because its synthesis method is fairly straightforward as compared with the synthesis of other QAMs.^27–28^ The synthesis of QADM involves a modified Menschutkin reaction in which a tertiary amine group is reacted with an organo-halide, and the reaction products are generated at virtually quantitative amounts and require minimal purification.^27, 28^ In addition, as a dimethacrylate, QADM can be beneficial as compared as quaternary ammonium monomethacrylates. QADM has reactive groups on both ends of the molecule, which can be incorporated into the resin matrix with less of a negative effect on the mechanical and physical properties of the composite.^27, 28^ In contrast, a quaternary ammonium monomethacrylate has only one reactive group and may weaken the resin matrix when incorporated. Furthermore, QADM is compatible with common dental dimethacrylate monomers and is expected to have minimal monomer leachability, compared with other quaternary ammonium salts. The detailed antimicrobial mechanism of quaternary ammonium remains to be fully understood; however, quaternary ammonium materials appear to cause bacterial lysis by binding to the cell membrane and causing cytoplasmic leakage.^25^ This leakage is stimulated when the negatively charged bacterial cell contacts the positively charged (N^+^) sites of the quaternary ammonium resin. This disturbs the electrical balance of the cell membrane, and the bacterium then ruptures under its own osmotic pressure. The results of the present study involving water-ageing for 1 year showed that such an antibacterial mechanism indeed has a long term and durable inhibitory effect on oral biofilm growth.

Ag is another effective and widely used antibacterial agent with a strong toxicity to a wide range of microorganisms.^52^ Its antimicrobial mechanism appears to involve Ag ions interacting with and inactivating the vital enzymes of bacteria, causing the DNA in the bacteria to lose its replication ability, leading to cell death.^52^ Although the present study indicates that the NAg in the composite possesses antibacterial activity even after 12 months of water-ageing, further studies are needed to investigate how long the antibacterial effect of NAg resin lasts and whether the antibacterial potency decreases after 2 or 3 years. Furthermore, the results of the present study showed that the use of two antibacterial agents (QADM+NAg) achieved a significantly stronger antibacterial potency than using either single agent, and the activity of the dual agents was maintained after water-ageing for 1 year. This was achieved because both QADM and silver, through TBAEMA's reactive methacrylate groups, which copolymerize with the resin matrix.^45^ These results suggest that copolymerization with the resin matrix and the use of dual agents appear to be promising approaches to maximize the antibacterial function of the resin without compromising its mechanical properties or long-term durability.

In addition to the QADM and NAg incorporation reducing biofilm acids and combating caries, NACP incorporation may also contribute to the inhibition of caries. A previous study has shown that a NACP composite can release Ca and P ions and neutralize an acid, rapidly increasing the pH of an acidic solution from a pH of 4 to one above 6.^53^ In contrast, commercial restorative controls fail to raise the pH.^53^ Other studies have shown that the NACP composite remineralizes enamel lesions^38^ and inhibits secondary caries at the composite-enamel margins in the oral environment, in a human *in situ* model.^54^ The NACP composite in these previous studies had no antibacterial agent in the composite. In the present study, incorporating both QADM and NAg into the NACP composite achieved double benefits: remineralization capability due to NACP and anti-biofilm activity due to QADM and NAg. Therefore, we expect that its caries-inhibition efficacy will be further enhanced when compared with NACP alone. Further studies are needed to compare the inhibition of secondary caries at the tooth-restoration margins by NACP *vs* NACP+QADM+NAg in a human *in situ* model.

In addition to the inhibition of secondary caries, another potential application of the novel NACP+QADM+NAg composite is in Class V restorations of root caries. The need for Class V restorations has increased as the world population ages.^55^ Gingival recession exposes more tooth roots, which have a higher solubility and are less resistant to biofilm acids than tooth enamel.^56^ In addition, reduced saliva flow tends to increase biofilm and plaque buildup.^57^ Indeed, root caries increase from 7% among young people to 56% in seniors.^58^ Class V restorations with subgingival restorative margins have pockets for bacterial growth that are difficult to clean and can lead to periodontitis.^59^ Therefore, there is a need to develop novel bioactive restoratives for Class V lesions that are antibacterial to inhibit cariogenic biofilms as well as periodontal pathogens in cases with subgingival margins. The antibacterial function of the NACP+QADM+NAg composite, as well as the ability of NACP to neutralize acids and to raise the pH above 6 (ref. 53) and to remineralize and protect the surrounding tooth structures,^38, 54^ may be beneficial and promising for Class V restorations. Further studies are needed to explore various clinical applications of the NACP+QADM+NAg composite.

## Conclusions

A novel NACP composite containing QADM and NAg was developed that possesses strong antibacterial capability that was maintained after 12 months of water-ageing. Flexural strength and elastic modulus of the bioactive composite after 12 months of water-ageing matched those of a commercial control composite without antibacterial properties. Incorporation of QADM or NAg into the NACP composite greatly reduced the oral microcosm biofilm viability, metabolic activity, CFUs and lactic acid production. Adding dual agents (QADM+NAg) in the same composite resulted in a significantly stronger antibacterial capability than using QADM or NAg alone. The antibacterial results were not significantly different after water-ageing for 12 months, compared with 1 day, thus indicating a long-term durability. The durable antibacterial and mechanical properties, plus the calcium and phosphate ion release and acid neutralization reported previously for remineralization, indicate that the novel NACP-QADM-NAg composites may be useful in dental restorations to combat caries.
